# Crystal structure of (4-hy­droxy­piperidin-1-yl)[4-(tri­fluoro­meth­yl)phen­yl]methanone

**DOI:** 10.1107/S205698901501765X

**Published:** 2015-09-26

**Authors:** B. K. Revathi, D. Reuben Jonathan, K. Kalai Sevi, K. Dhanalakshmi, G. Usha

**Affiliations:** a PG and Research Department of Physics, Queen Mary’s College, Chennai-4, Tamilnadu, India; b Department of Chemistry, Madras Christian College, Chennai-59, India; c SCRI, Anna Hospital Campus, Chennai-106, Tamilnadu, India; d Anna Siddha Medical College, Chennai-106, Tamilnadu, India

**Keywords:** crystal structure, piperdine derivative, hydrogen bonding

## Abstract

The title compound, C_13_H_14_NO_2_F_3_, crystallises with two mol­ecules, *A* and *B*, in the asymmetric unit, with similar conformations. The dihedral angles between the piperidine and phenyl rings are 83.76 (2) and 75.23 (2)° in mol­ecules *A* and *B*, respectively. The bond-angle sums around the N atoms [359.1 and 359.7° for mol­ecules *A* and *B*, respectively] indicate *sp* ^2^ hybridization for these atoms. In the crystal, O—H⋯O hydrogen bonds link the mol­ecules into separate [100] chains of *A* and *B* mol­ecules. The chains are cross-linked by C—H⋯O inter­actions, generating alternating (001) sheets of *A* and *B* mol­ecules.

## Related literature   

For the synthesis, see: Revathi *et al.* (2015 ▸). For the biological activities of piperdine derivatives, see: Ramalingan *et al.* (2004 ▸); Ramachandran *et al.* (2011 ▸); Lee *et al.* (2001 ▸); Parthiban *et al.* (2005 ▸). For a related structure, see: Prathebha *et al.* (2015 ▸).
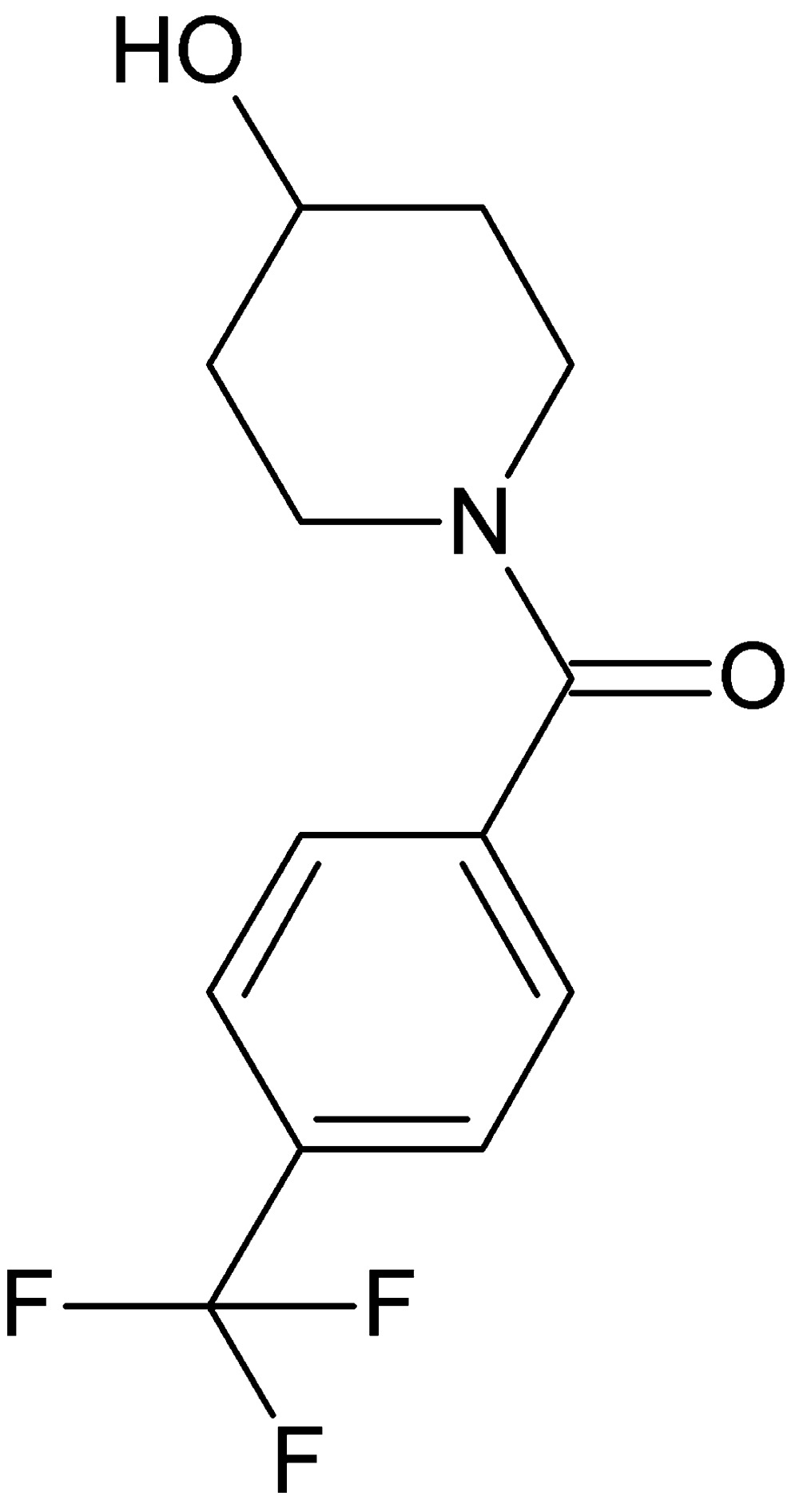


## Experimental   

### Crystal data  

C_13_H_14_F_3_NO_2_
*M* *_r_* = 273.25
Orthorhombic, 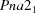

*a* = 16.1328 (14) Å
*b* = 6.8283 (6) Å
*c* = 23.017 (2) Å
*V* = 2535.5 (4) Å^3^
*Z* = 8
Mo *K*α radiation
μ = 0.13 mm^−1^
*T* = 293 K
0.35 × 0.30 × 0.25 mm

### Data collection  

Bruker Kappa APEXII CCD diffractometer
Absorption correction: multi-scan (*SADABS*; Bruker, 2004 ▸) *T*_min_ = 0.957, *T*_max_ = 0.969
26641 measured reflections
4823 independent reflections
2950 reflections with *I* > 2σ(*I*)
*R*_int_ = 0.046

### Refinement  

*R*[*F* ^2^ > 2σ(*F* ^2^)] = 0.054
*wR*(*F* ^2^) = 0.190
*S* = 1.07
4823 reflections
343 parameters
1 restraint
H atoms treated by a mixture of independent and constrained refinement
Δρ_max_ = 0.27 e Å^−3^
Δρ_min_ = −0.26 e Å^−3^

### 

Data collection: *APEX2* (Bruker, 2004 ▸); cell refinement: *SAINT* (Bruker, 2004 ▸); data reduction: *SAINT*; program(s) used to solve structure: *SHELXS97* (Sheldrick, 2008 ▸); program(s) used to refine structure: *SHELXL97* (Sheldrick, 2008 ▸); molecular graphics: *ORTEP-3 for Windows* (Farrugia, 2012 ▸); software used to prepare material for publication: *SHELXL97*.

## Supplementary Material

Crystal structure: contains datablock(s) I, New_Global_Publ_Block. DOI: 10.1107/S205698901501765X/hb7494sup1.cif

Structure factors: contains datablock(s) I. DOI: 10.1107/S205698901501765X/hb7494Isup2.hkl

Supporting information file. DOI: 10.1107/S205698901501765X/hb7494Isup3.cml

**Fig. 1.**
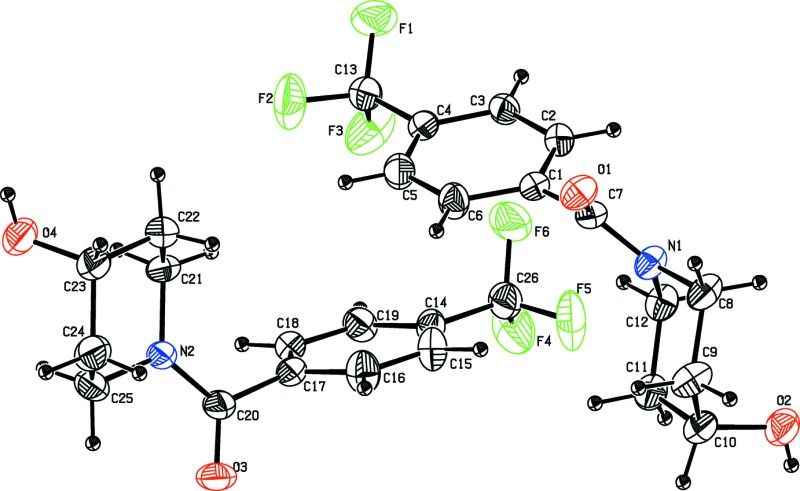
The molecular structure of the title compound, with displacement ellipsoids drawn at the 30% probability level.

. DOI: 10.1107/S205698901501765X/hb7494fig1.tif
The mol­ecular structure of the title compound, with displacement ellipsoids drawn at the 30% probability level.

**Fig. 2.**
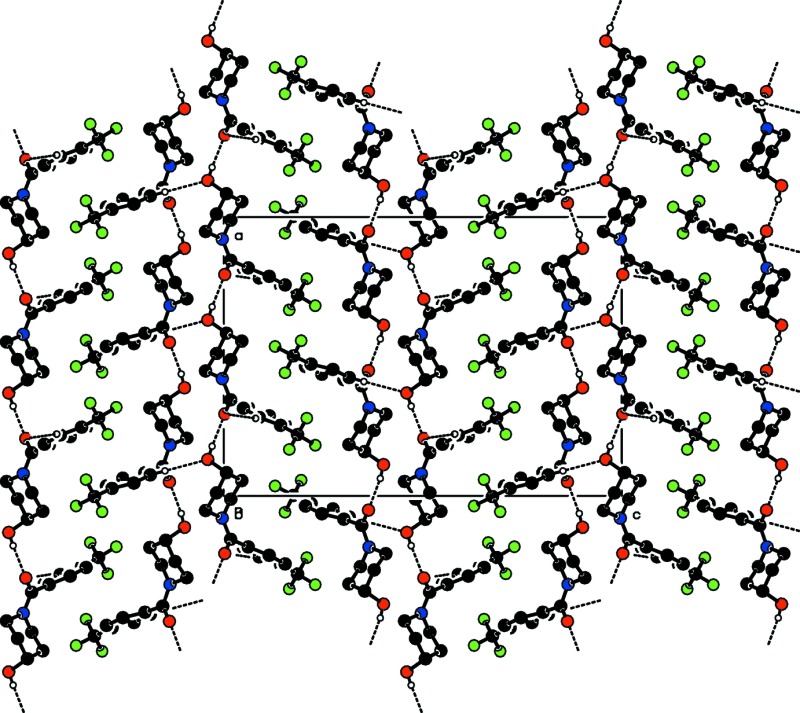
The packing of the molecules in the crystal structure. The dashed lines indicate the hydrogen bonds.

. DOI: 10.1107/S205698901501765X/hb7494fig2.tif
The packing of the mol­ecules in the crystal structure. The dashed lines indicate the hydrogen bonds.

CCDC reference: 1425996

Additional supporting information:  crystallographic information; 3D view; checkCIF report

## Figures and Tables

**Table 1:** Hydrogen-bond geometry (, )

*D*H*A*	*D*H	H*A*	*D* *A*	*D*H*A*
O2H1*A*O1^i^	0.82	2.01	2.819(4)	169
O4H3O3^ii^	0.82	1.96	2.775(5)	173
C3H9O1^iii^	0.93	2.55	3.367(6)	147
C18H21O2^iv^	0.93	2.58	3.445(7)	156

Symmetry codes: (i) 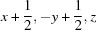; (ii) 
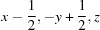
; (iii) 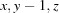; (iv) 
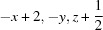
.

## supplementary crystallographic information

### S1. Comment

The stucture of the title compound, (I), is shown below. Dimensions are available in the archived CIF.

The motivation for the biological trial arises as piperidine derivatives are an important class of heterocyclic compounds with potent pharmacological/ biological activities (Ramalingan *et al.*, 2004; Ramachandran *et al.*, 2011). Heterocycles with piperidine sub-structures are being used as synthons in the construction of alkaloid natural products (Lee *et al.*, 2001). Piperidine derivatives exhibit a broad-spectrum of biological activities such as anti- bacterial and anti-cancer (Parthiban *et al.*, 2005).

In the title compound,the C—N distances of piperidine ring in molecule A(C8—C12/N1) & B(C21—C25/N2) are in the range[1.318 (6)–1.462 (6) Å] and [1.329 (6)–1.458 (6) Å] are in good agreement with values of a similar reported structure (Revathi *et al.*, 2015). The C—O distance in molecule A & B are [1.242 (6) Å & 1.227 (6) Å], it indicates double bond character and are comparable with the value reported previously (Prathebha *et al.*, 2015). The dihedral angle between piperidine ring and phenyl ring in molecule A & B are 83.76 (2)° and 75.23 (2)°. The Piperidine rings are in equatorial (eq) orientation with the corresponding phenyl rings. The sum of the bond angles around the N1 & N2 atoms are [359.1 (4)° and 359.7 (4)°, respectively], shows sP^2^ hybridization of the atoms. The piperidine ring in the molecule A adopts a chair conformation with puckering parameters of q2 = 0.051 (5) Å, Phi2 = 138.01°, q3 = -0.537 (6) Å, QT = 0.539 (6) Å and theta2 = 174.53 (5)°. The piperidine ring in the molecule B adopts a chair conformation with puckering parameters of q2 = 0.048 (6) Å, Phi2 = 159.33°, q3 = -0.561 (6) Å, QT = 0.563 (6) Å and theta2 = 175.12 (6)°.

### S2. Experimental

The title compound was synthesized following a published procedure (Revathi *et al.*, 2015). In a 250 ml round-bottomed flask, 100 ml of ethylmethylketone was added to 4-hydroxypiperidiene (0.03 mol) and stirred at room temperature. After a span of about 5 min, triethylamine (0.03 mol) was added and the mixture was stirred for a time frame of 10 min. 4-Trifloromethylbenzoyl chloride (0.03 mol) was added and the reaction mixture was stirred at room temperature for about 2 h. A white precipitate of triethylammonium chloride was produced, which was filtered and the filtrate was evaporated to get the crude product. Two recrystallizations from ethylmethylketone solution gave colourless blocks of the title compound (yield: 87%).

### S3. Refinement

Hydrogen atoms other then hydroxy H atoms were positioned geometrically and treated as riding on their parent atoms and hydroxy H-atoms were located from difference Fourier maps and refined with,*C*—H distance of 0.93–0.98 Å, with *U*_iso_(H)= 1.5 *U*_eq_(c-methyl), *U*_iso_(H)= 1.2Ueq(C,*O*) for other H atom.

### Crystal data

**Table:**

C_13_H_14_F_3_NO_2_	*F*(000) = 1136
*M**_r_* = 273.25	*D*_x_ = 1.432 Mg m^−^^3^
Orthorhombic, *P**n**a*2_1_	Mo *K*α radiation, λ = 0.71073 Å
Hall symbol: P 2c -2n	θ = 1.8–25.7°
*a* = 16.1328 (14) Å	µ = 0.13 mm^−^^1^
*b* = 6.8283 (6) Å	*T* = 293 K
*c* = 23.017 (2) Å	Block, colorless
*V* = 2535.5 (4) Å^3^	0.35 × 0.30 × 0.25 mm
*Z* = 8	

### Data collection

**Table:**

Bruker Kappa APEXII CCD diffractometer	4823 independent reflections
Radiation source: fine-focus sealed tube	2950 reflections with *I* > 2σ(*I*)
Graphite monochromator	*R*_int_ = 0.046
ω and φ scans	θ_max_ = 25.7°, θ_min_ = 2.5°
Absorption correction: multi-scan (*SADABS*; Bruker, 2004)	*h* = −19→19
*T*_min_ = 0.957, *T*_max_ = 0.969	*k* = −8→7
26641 measured reflections	*l* = −28→28

### Refinement

**Table:**

Refinement on *F*^2^	Primary atom site location: structure-invariant direct methods
Least-squares matrix: full	Secondary atom site location: difference Fourier map
*R*[*F*^2^ > 2σ(*F*^2^)] = 0.054	Hydrogen site location: inferred from neighbouring sites
*wR*(*F*^2^) = 0.190	H atoms treated by a mixture of independent and constrained refinement
*S* = 1.07	*w* = 1/[σ^2^(*F*_o_^2^) + (0.0905*P*)^2^ + 1.4379*P*] where *P* = (*F*_o_^2^ + 2*F*_c_^2^)/3
4823 reflections	(Δ/σ)_max_ < 0.001
343 parameters	Δρ_max_ = 0.27 e Å^−^^3^
1 restraint	Δρ_min_ = −0.26 e Å^−^^3^

### Special details

**Table:**

Geometry. All e.s.d.'s (except the e.s.d. in the dihedral angle between two l.s. planes) are estimated using the full covariance matrix. The cell e.s.d.'s are taken into account individually in the estimation of e.s.d.'s in distances, angles and torsion angles; correlations between e.s.d.'s in cell parameters are only used when they are defined by crystal symmetry. An approximate (isotropic) treatment of cell e.s.d.'s is used for estimating e.s.d.'s involving l.s. planes.
Refinement. Refinement of *F*^2^ against ALL reflections. The weighted *R*-factor *wR* and goodness of fit *S* are based on *F*^2^, conventional *R*-factors *R* are based on *F*, with *F* set to zero for negative *F*^2^. The threshold expression of *F*^2^ > σ(*F*^2^) is used only for calculating *R*-factors(gt) *etc*. and is not relevant to the choice of reflections for refinement. *R*-factors based on *F*^2^ are statistically about twice as large as those based on *F*, and *R*- factors based on ALL data will be even larger.

### Fractional atomic coordinates and isotropic or equivalent isotropic displacement parameters (Å^2^)

**Table:**

	*x*	*y*	*z*	*U*_iso_*/*U*_eq_	
C10	1.0836 (3)	0.3171 (9)	0.0067 (2)	0.0622 (13)	
H1	1.1181	0.4072	0.0294	0.075*	
C9	1.0108 (3)	0.4279 (8)	−0.0181 (3)	0.0649 (14)	
H2A	0.9844	0.5021	0.0127	0.078*	
H2B	1.0308	0.5203	−0.0469	0.078*	
C8	0.9480 (3)	0.2956 (8)	−0.0457 (2)	0.0593 (13)	
H3A	0.9008	0.3719	−0.0587	0.071*	
H3B	0.9722	0.2316	−0.0793	0.071*	
C12	0.9885 (3)	0.0264 (7)	0.0177 (2)	0.0523 (11)	
H4A	1.0121	−0.0478	−0.0142	0.063*	
H4B	0.9673	−0.0657	0.0462	0.063*	
C11	1.0551 (3)	0.1530 (8)	0.0453 (2)	0.0580 (13)	
H5A	1.0338	0.2078	0.0812	0.070*	
H5B	1.1023	0.0714	0.0550	0.070*	
C7	0.8449 (3)	0.1543 (6)	0.0173 (2)	0.0448 (10)	
C1	0.8174 (3)	0.0056 (7)	0.06151 (19)	0.0450 (11)	
C2	0.8144 (3)	−0.1940 (7)	0.0494 (2)	0.0495 (11)	
H8	0.8348	−0.2411	0.0142	0.059*	
C3	0.7815 (3)	−0.3204 (7)	0.0891 (2)	0.0485 (11)	
H9	0.7780	−0.4530	0.0802	0.058*	
C4	0.7532 (3)	−0.2536 (7)	0.1427 (2)	0.0502 (12)	
C5	0.7564 (3)	−0.0557 (7)	0.1542 (2)	0.0606 (13)	
H11	0.7372	−0.0091	0.1897	0.073*	
C6	0.7873 (3)	0.0733 (7)	0.1142 (2)	0.0570 (13)	
H12	0.7881	0.2066	0.1224	0.068*	
C13	0.7202 (3)	−0.3921 (7)	0.1854 (2)	0.0589 (12)	
C23	0.6591 (3)	0.3211 (8)	0.3540 (2)	0.0612 (13)	
H14	0.6260	0.4154	0.3318	0.073*	
C22	0.6845 (3)	0.1537 (8)	0.3146 (2)	0.0602 (13)	
H15A	0.7085	0.2067	0.2794	0.072*	
H15B	0.6355	0.0795	0.3039	0.072*	
C21	0.7469 (3)	0.0165 (7)	0.3433 (2)	0.0577 (13)	
H16A	0.7206	−0.0528	0.3752	0.069*	
H16B	0.7661	−0.0795	0.3153	0.069*	
C25	0.7932 (3)	0.2784 (9)	0.4071 (3)	0.0730 (17)	
H17A	0.8421	0.3465	0.4209	0.088*	
H17B	0.7660	0.2179	0.4401	0.088*	
C24	0.7348 (3)	0.4220 (8)	0.3779 (3)	0.0737 (16)	
H18A	0.7179	0.5205	0.4058	0.088*	
H18B	0.7638	0.4878	0.3465	0.088*	
C20	0.8949 (3)	0.1163 (8)	0.3466 (2)	0.0552 (12)	
C17	0.9167 (3)	−0.0342 (7)	0.3019 (2)	0.0492 (11)	
C18	0.9246 (3)	−0.2314 (8)	0.3153 (2)	0.0548 (13)	
H21	0.9102	−0.2764	0.3521	0.066*	
C19	0.9537 (3)	−0.3609 (7)	0.2742 (2)	0.0574 (12)	
H22	0.9605	−0.4926	0.2833	0.069*	
C14	0.9728 (3)	−0.2926 (7)	0.2193 (2)	0.0528 (12)	
C15	0.9639 (3)	−0.1019 (8)	0.2054 (2)	0.0688 (15)	
H24	0.9766	−0.0586	0.1681	0.083*	
C16	0.9363 (4)	0.0281 (8)	0.2465 (2)	0.0688 (15)	
H25	0.9306	0.1597	0.2369	0.083*	
C26	1.0021 (3)	−0.4353 (8)	0.1751 (2)	0.0635 (14)	
N1	0.9207 (2)	0.1482 (6)	−0.00380 (16)	0.0494 (10)	
N2	0.8169 (2)	0.1289 (7)	0.36505 (17)	0.0552 (11)	
O2	1.13070 (19)	0.2418 (6)	−0.04018 (18)	0.0719 (11)	
H1A	1.1781	0.2194	−0.0292	0.108*	
O1	0.79406 (18)	0.2827 (5)	0.00270 (16)	0.0574 (9)	
O4	0.6124 (2)	0.2524 (6)	0.40139 (18)	0.0712 (10)	
H3	0.5629	0.2553	0.3932	0.107*	
O3	0.9492 (2)	0.2285 (7)	0.36352 (19)	0.0842 (14)	
F1	0.6669 (2)	−0.5173 (6)	0.16460 (16)	0.1029 (13)	
F2	0.6821 (3)	−0.3078 (6)	0.22995 (16)	0.1089 (14)	
F3	0.7790 (2)	−0.5065 (6)	0.20861 (18)	0.1101 (14)	
F4	1.0566 (2)	−0.5623 (6)	0.19448 (16)	0.1038 (13)	
F5	1.0359 (3)	−0.3537 (6)	0.12899 (16)	0.1165 (15)	
F6	0.9416 (2)	−0.5492 (6)	0.15396 (18)	0.1028 (12)	

### Atomic displacement parameters (Å^2^)

**Table:**

	*U*^11^	*U*^22^	*U*^33^	*U*^12^	*U*^13^	*U*^23^
C10	0.044 (3)	0.079 (3)	0.063 (3)	−0.006 (3)	0.005 (3)	0.000 (3)
C9	0.047 (3)	0.063 (3)	0.085 (4)	−0.002 (2)	0.001 (3)	0.020 (3)
C8	0.041 (3)	0.080 (3)	0.057 (3)	0.003 (2)	0.001 (2)	0.028 (3)
C12	0.046 (2)	0.054 (3)	0.057 (3)	0.014 (2)	0.003 (2)	0.010 (2)
C11	0.044 (3)	0.080 (3)	0.050 (3)	0.010 (2)	−0.002 (2)	0.011 (3)
C7	0.037 (2)	0.048 (2)	0.049 (3)	−0.003 (2)	−0.003 (2)	0.000 (2)
C1	0.039 (2)	0.049 (3)	0.047 (3)	−0.005 (2)	0.002 (2)	−0.002 (2)
C2	0.052 (3)	0.054 (3)	0.042 (3)	−0.001 (2)	0.004 (2)	−0.001 (2)
C3	0.054 (3)	0.043 (2)	0.048 (3)	−0.005 (2)	0.003 (2)	−0.007 (2)
C4	0.042 (3)	0.064 (3)	0.044 (3)	−0.008 (2)	0.001 (2)	0.001 (2)
C5	0.071 (3)	0.057 (3)	0.055 (3)	−0.012 (3)	0.015 (3)	−0.008 (3)
C6	0.069 (3)	0.047 (3)	0.054 (3)	−0.003 (2)	0.013 (2)	−0.008 (2)
C13	0.061 (3)	0.063 (3)	0.052 (3)	−0.008 (3)	0.002 (2)	0.001 (3)
C23	0.048 (3)	0.077 (3)	0.058 (3)	−0.003 (3)	0.001 (3)	0.001 (3)
C22	0.049 (3)	0.075 (3)	0.056 (3)	−0.008 (2)	−0.001 (2)	−0.009 (3)
C21	0.041 (3)	0.065 (3)	0.067 (3)	−0.014 (2)	0.002 (2)	−0.014 (3)
C25	0.046 (3)	0.102 (4)	0.071 (4)	0.003 (3)	−0.005 (3)	−0.044 (4)
C24	0.062 (3)	0.067 (3)	0.093 (4)	−0.009 (3)	0.019 (3)	−0.027 (3)
C20	0.038 (2)	0.072 (3)	0.056 (3)	0.000 (2)	−0.004 (2)	−0.016 (3)
C17	0.034 (2)	0.061 (3)	0.052 (3)	0.000 (2)	0.002 (2)	−0.008 (2)
C18	0.050 (3)	0.068 (3)	0.046 (3)	−0.013 (2)	0.004 (2)	−0.006 (2)
C19	0.065 (3)	0.054 (3)	0.053 (3)	−0.003 (2)	−0.002 (2)	−0.002 (3)
C14	0.045 (2)	0.066 (3)	0.047 (3)	0.008 (2)	0.006 (2)	−0.005 (2)
C15	0.080 (4)	0.078 (4)	0.048 (3)	0.008 (3)	0.017 (3)	0.001 (3)
C16	0.089 (4)	0.055 (3)	0.062 (3)	0.008 (3)	0.013 (3)	0.002 (3)
C26	0.057 (3)	0.071 (3)	0.063 (3)	0.010 (3)	0.003 (3)	−0.012 (3)
N1	0.040 (2)	0.058 (2)	0.050 (2)	0.0036 (18)	0.0025 (17)	0.0156 (19)
N2	0.034 (2)	0.076 (3)	0.056 (3)	−0.0026 (18)	0.0028 (17)	−0.026 (2)
O2	0.0471 (19)	0.108 (3)	0.061 (2)	0.003 (2)	0.0028 (17)	0.005 (2)
O1	0.0420 (18)	0.059 (2)	0.071 (2)	0.0078 (15)	0.0006 (16)	0.0096 (17)
O4	0.0499 (19)	0.096 (3)	0.067 (3)	−0.0026 (19)	0.0084 (18)	0.006 (2)
O3	0.0412 (18)	0.107 (3)	0.105 (3)	−0.013 (2)	−0.0003 (19)	−0.053 (3)
F1	0.110 (3)	0.116 (3)	0.082 (2)	−0.065 (2)	−0.004 (2)	0.014 (2)
F2	0.155 (4)	0.095 (2)	0.076 (2)	−0.016 (2)	0.057 (2)	0.002 (2)
F3	0.087 (2)	0.127 (3)	0.116 (3)	0.004 (2)	0.009 (2)	0.069 (3)
F4	0.097 (3)	0.119 (3)	0.096 (3)	0.053 (2)	−0.008 (2)	−0.029 (2)
F5	0.168 (4)	0.109 (3)	0.072 (2)	0.004 (3)	0.060 (3)	−0.012 (2)
F6	0.079 (2)	0.119 (3)	0.110 (3)	0.003 (2)	0.001 (2)	−0.059 (3)

### Geometric parameters (Å, º)

**Table:**

C10—O2	1.417 (7)	C23—C24	1.508 (7)
C10—C11	1.501 (8)	C23—C22	1.514 (7)
C10—C9	1.509 (7)	C23—H14	0.9800
C10—H1	0.9800	C22—C21	1.526 (7)
C9—C8	1.499 (8)	C22—H15A	0.9700
C9—H2A	0.9700	C22—H15B	0.9700
C9—H2B	0.9700	C21—N2	1.455 (6)
C8—N1	1.462 (6)	C21—H16A	0.9700
C8—H3A	0.9700	C21—H16B	0.9700
C8—H3B	0.9700	C25—N2	1.457 (6)
C12—N1	1.460 (6)	C25—C24	1.516 (9)
C12—C11	1.519 (7)	C25—H17A	0.9700
C12—H4A	0.9700	C25—H17B	0.9700
C12—H4B	0.9700	C24—H18A	0.9700
C11—H5A	0.9700	C24—H18B	0.9700
C11—H5B	0.9700	C20—O3	1.228 (6)
C7—O1	1.247 (5)	C20—N2	1.330 (6)
C7—N1	1.316 (6)	C20—C17	1.496 (7)
C7—C1	1.505 (6)	C17—C16	1.381 (8)
C1—C6	1.386 (6)	C17—C18	1.387 (7)
C1—C2	1.392 (7)	C18—C19	1.378 (7)
C2—C3	1.364 (7)	C18—H21	0.9300
C2—H8	0.9300	C19—C14	1.380 (7)
C3—C4	1.394 (7)	C19—H22	0.9300
C3—H9	0.9300	C14—C15	1.349 (7)
C4—C5	1.378 (7)	C14—C26	1.486 (7)
C4—C13	1.464 (7)	C15—C16	1.372 (7)
C5—C6	1.368 (7)	C15—H24	0.9300
C5—H11	0.9300	C16—H25	0.9300
C6—H12	0.9300	C26—F4	1.313 (6)
C13—F1	1.304 (6)	C26—F5	1.317 (7)
C13—F2	1.327 (6)	C26—F6	1.339 (6)
C13—F3	1.339 (6)	O2—H1A	0.8200
C23—O4	1.407 (6)	O4—H3	0.8200
			
O2—C10—C11	110.1 (5)	C24—C23—H14	109.4
O2—C10—C9	108.1 (5)	C22—C23—H14	109.4
C11—C10—C9	111.1 (4)	C23—C22—C21	112.6 (4)
O2—C10—H1	109.2	C23—C22—H15A	109.1
C11—C10—H1	109.2	C21—C22—H15A	109.1
C9—C10—H1	109.2	C23—C22—H15B	109.1
C8—C9—C10	112.6 (5)	C21—C22—H15B	109.1
C8—C9—H2A	109.1	H15A—C22—H15B	107.8
C10—C9—H2A	109.1	N2—C21—C22	109.7 (4)
C8—C9—H2B	109.1	N2—C21—H16A	109.7
C10—C9—H2B	109.1	C22—C21—H16A	109.7
H2A—C9—H2B	107.8	N2—C21—H16B	109.7
N1—C8—C9	109.8 (4)	C22—C21—H16B	109.7
N1—C8—H3A	109.7	H16A—C21—H16B	108.2
C9—C8—H3A	109.7	N2—C25—C24	108.8 (5)
N1—C8—H3B	109.7	N2—C25—H17A	109.9
C9—C8—H3B	109.7	C24—C25—H17A	109.9
H3A—C8—H3B	108.2	N2—C25—H17B	109.9
N1—C12—C11	110.3 (4)	C24—C25—H17B	109.9
N1—C12—H4A	109.6	H17A—C25—H17B	108.3
C11—C12—H4A	109.6	C23—C24—C25	111.7 (5)
N1—C12—H4B	109.6	C23—C24—H18A	109.3
C11—C12—H4B	109.6	C25—C24—H18A	109.3
H4A—C12—H4B	108.1	C23—C24—H18B	109.3
C10—C11—C12	113.2 (4)	C25—C24—H18B	109.3
C10—C11—H5A	108.9	H18A—C24—H18B	107.9
C12—C11—H5A	108.9	O3—C20—N2	122.3 (4)
C10—C11—H5B	108.9	O3—C20—C17	118.5 (4)
C12—C11—H5B	108.9	N2—C20—C17	119.1 (4)
H5A—C11—H5B	107.7	C16—C17—C18	118.8 (5)
O1—C7—N1	122.3 (4)	C16—C17—C20	118.5 (5)
O1—C7—C1	117.6 (4)	C18—C17—C20	122.4 (5)
N1—C7—C1	120.1 (4)	C19—C18—C17	120.2 (5)
C6—C1—C2	119.3 (4)	C19—C18—H21	119.9
C6—C1—C7	118.1 (4)	C17—C18—H21	119.9
C2—C1—C7	122.4 (4)	C18—C19—C14	119.2 (5)
C3—C2—C1	119.9 (5)	C18—C19—H22	120.4
C3—C2—H8	120.0	C14—C19—H22	120.4
C1—C2—H8	120.0	C15—C14—C19	121.3 (5)
C2—C3—C4	120.9 (4)	C15—C14—C26	120.3 (5)
C2—C3—H9	119.5	C19—C14—C26	118.4 (5)
C4—C3—H9	119.5	C14—C15—C16	119.7 (5)
C5—C4—C3	118.6 (5)	C14—C15—H24	120.2
C5—C4—C13	121.2 (5)	C16—C15—H24	120.2
C3—C4—C13	120.2 (4)	C15—C16—C17	120.8 (5)
C6—C5—C4	121.1 (5)	C15—C16—H25	119.6
C6—C5—H11	119.5	C17—C16—H25	119.6
C4—C5—H11	119.5	F4—C26—F5	106.0 (5)
C5—C6—C1	120.1 (5)	F4—C26—F6	103.2 (4)
C5—C6—H12	119.9	F5—C26—F6	104.7 (5)
C1—C6—H12	119.9	F4—C26—C14	114.5 (5)
F1—C13—F2	105.2 (5)	F5—C26—C14	114.0 (5)
F1—C13—F3	103.4 (4)	F6—C26—C14	113.4 (4)
F2—C13—F3	105.8 (4)	C7—N1—C12	126.1 (4)
F1—C13—C4	114.7 (4)	C7—N1—C8	120.0 (4)
F2—C13—C4	114.0 (4)	C12—N1—C8	113.0 (4)
F3—C13—C4	112.8 (4)	C20—N2—C21	126.2 (4)
O4—C23—C24	107.6 (4)	C20—N2—C25	120.3 (4)
O4—C23—C22	110.9 (5)	C21—N2—C25	113.2 (4)
C24—C23—C22	110.1 (4)	C10—O2—H1A	109.5
O4—C23—H14	109.4	C23—O4—H3	109.5
			
O2—C10—C9—C8	70.2 (6)	O3—C20—C17—C18	106.8 (6)
C11—C10—C9—C8	−50.8 (7)	N2—C20—C17—C18	−75.7 (6)
C10—C9—C8—N1	55.5 (6)	C16—C17—C18—C19	1.8 (7)
O2—C10—C11—C12	−71.0 (5)	C20—C17—C18—C19	−172.8 (4)
C9—C10—C11—C12	48.7 (6)	C17—C18—C19—C14	−1.7 (7)
N1—C12—C11—C10	−51.7 (6)	C18—C19—C14—C15	0.5 (8)
O1—C7—C1—C6	−55.1 (6)	C18—C19—C14—C26	−178.5 (4)
N1—C7—C1—C6	123.6 (5)	C19—C14—C15—C16	0.6 (8)
O1—C7—C1—C2	119.0 (5)	C26—C14—C15—C16	179.5 (5)
N1—C7—C1—C2	−62.2 (6)	C14—C15—C16—C17	−0.5 (9)
C6—C1—C2—C3	0.4 (7)	C18—C17—C16—C15	−0.8 (8)
C7—C1—C2—C3	−173.7 (4)	C20—C17—C16—C15	174.1 (5)
C1—C2—C3—C4	−2.2 (7)	C15—C14—C26—F4	137.5 (5)
C2—C3—C4—C5	2.3 (7)	C19—C14—C26—F4	−43.5 (7)
C2—C3—C4—C13	−178.3 (4)	C15—C14—C26—F5	15.3 (8)
C3—C4—C5—C6	−0.6 (8)	C19—C14—C26—F5	−165.7 (5)
C13—C4—C5—C6	180.0 (5)	C15—C14—C26—F6	−104.4 (6)
C4—C5—C6—C1	−1.1 (8)	C19—C14—C26—F6	74.6 (6)
C2—C1—C6—C5	1.2 (7)	O1—C7—N1—C12	168.7 (4)
C7—C1—C6—C5	175.6 (4)	C1—C7—N1—C12	−10.0 (7)
C5—C4—C13—F1	132.1 (5)	O1—C7—N1—C8	0.2 (7)
C3—C4—C13—F1	−47.2 (7)	C1—C7—N1—C8	−178.5 (4)
C5—C4—C13—F2	10.8 (7)	C11—C12—N1—C7	−111.8 (5)
C3—C4—C13—F2	−168.6 (5)	C11—C12—N1—C8	57.4 (5)
C5—C4—C13—F3	−109.9 (6)	C9—C8—N1—C7	110.3 (5)
C3—C4—C13—F3	70.7 (6)	C9—C8—N1—C12	−59.5 (6)
O4—C23—C22—C21	−67.9 (5)	O3—C20—N2—C21	173.3 (5)
C24—C23—C22—C21	51.1 (6)	C17—C20—N2—C21	−4.0 (8)
C23—C22—C21—N2	−53.0 (5)	O3—C20—N2—C25	−0.5 (9)
O4—C23—C24—C25	67.5 (6)	C17—C20—N2—C25	−177.9 (5)
C22—C23—C24—C25	−53.5 (6)	C22—C21—N2—C20	−115.5 (6)
N2—C25—C24—C23	57.8 (6)	C22—C21—N2—C25	58.7 (6)
O3—C20—C17—C16	−67.8 (7)	C24—C25—N2—C20	113.5 (5)
N2—C20—C17—C16	109.6 (6)	C24—C25—N2—C21	−61.1 (6)

### Hydrogen-bond geometry (Å, º)

**Table:**

*D*—H···*A*	*D*—H	H···*A*	*D*···*A*	*D*—H···*A*
O2—H1*A*···O1^i^	0.82	2.01	2.819 (4)	169
O4—H3···O3^ii^	0.82	1.96	2.775 (5)	173
C3—H9···O1^iii^	0.93	2.55	3.367 (6)	147
C18—H21···O2^iv^	0.93	2.58	3.445 (7)	156

Symmetry codes: (i) *x*+1/2, −*y*+1/2, *z*; (ii) *x*−1/2, −*y*+1/2, *z*; (iii) *x*, *y*−1, *z*; (iv) −*x*+2, −*y*, *z*+1/2.
