# Supplementary figures and images for: Crystal structure of (4-hy­droxy­piperidin-1-yl)[4-(tri­fluoro­meth­yl)phen­yl]methanone
Source: Acta Crystallogr E Crystallogr Commun. 2015 Sep 26;71(Pt 10):o790–1. doi: 10.1107/S205698901501765X (PMC4647357; doi:10.1107/S205698901501765X)

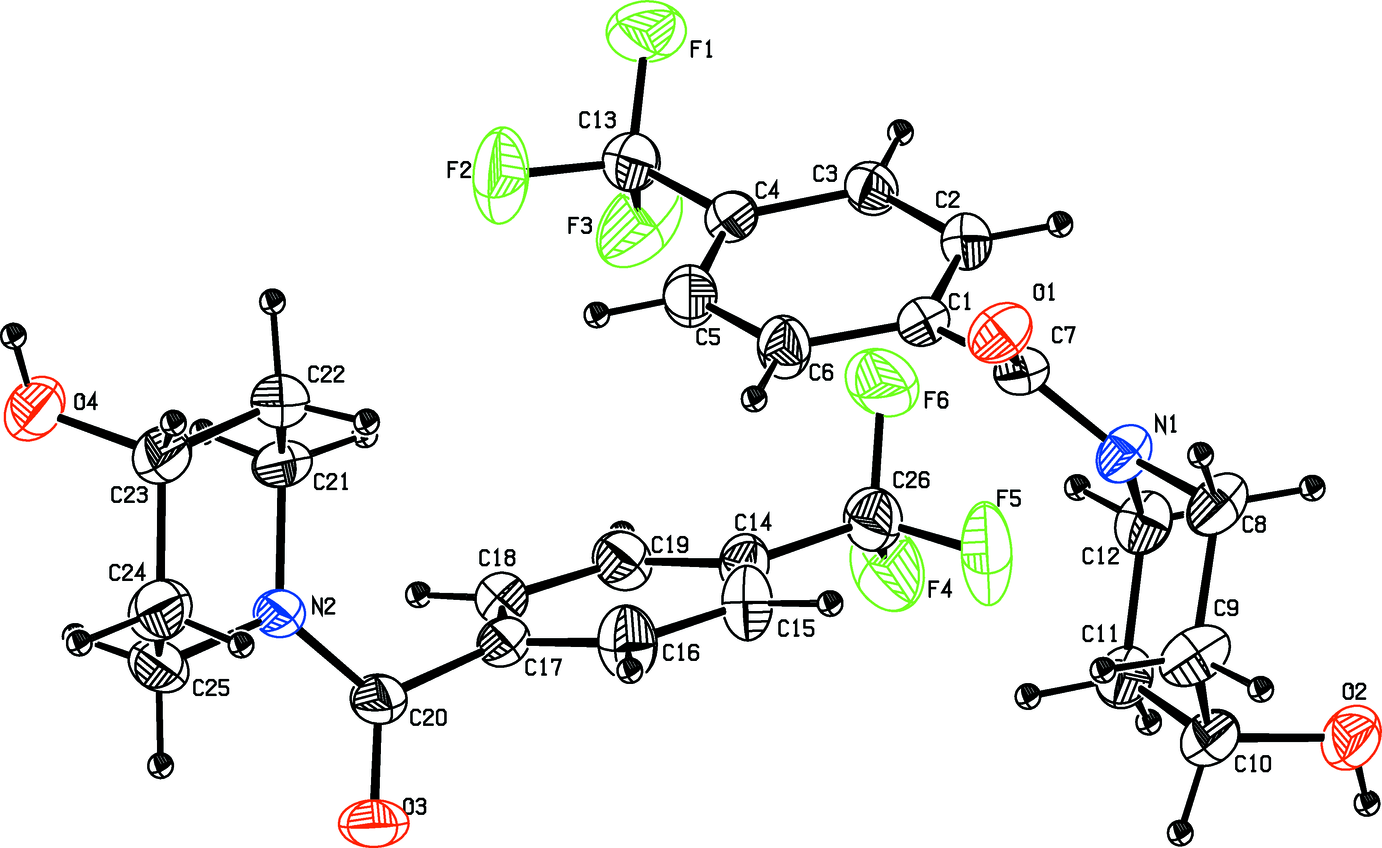

Supplement: Supplementary file 4 [file e-71-0o790-fig1.tif]

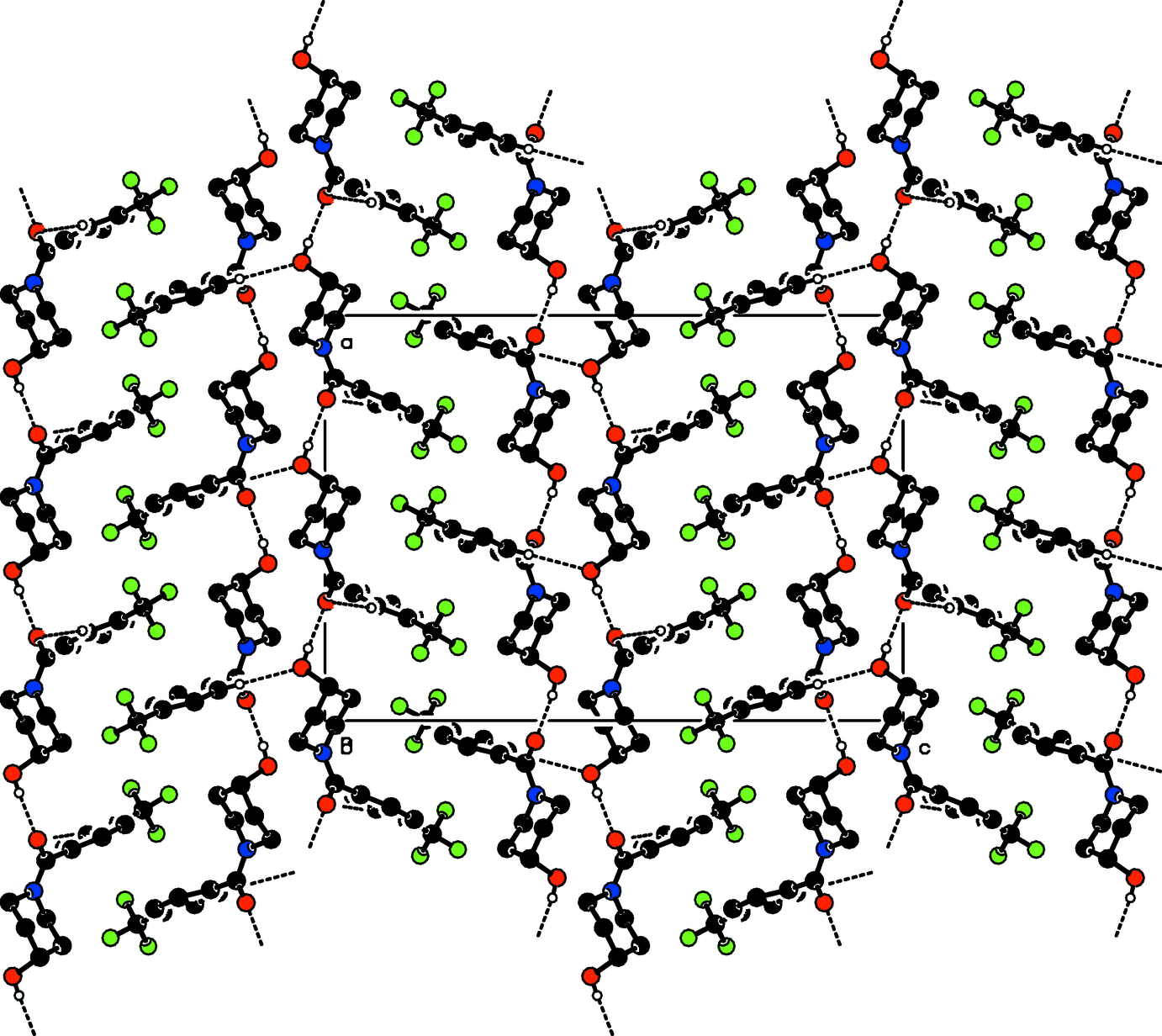

Supplement: Supplementary file 5 [file e-71-0o790-fig2.tif]
